# Nanoscale Clustering in an Additively Manufactured Zr-Based Metallic Glass Evaluated by Atom Probe Tomography

**DOI:** 10.3390/nano13081341

**Published:** 2023-04-12

**Authors:** Inga K. Goetz, Janis A. Sälker, Marcus Hans, Björgvin Hjörvarsson, Jochen M. Schneider

**Affiliations:** 1 Department of Physics and Astronomy, Materials Physics, Uppsala University, Box 530, SE-75121 Uppsala, Sweden; bjorgvin.hjorvarsson@physics.uu.se; 2 Materials Chemistry, RWTH Aachen University, Kopernikusstr. 10, D-52074 Aachen, Germany

**Keywords:** bulk metallic glasses, laser powder bed fusion, spatial isotope distribution, random spatial configuration, onset of devitrification

## Abstract

Composition analysis at the nm-scale, marking the onset of clustering in bulk metallic glasses, can aid the understanding and further optimization of additive manufacturing processes. By atom probe tomography, it is challenging to differentiate nm-scale segregations from random fluctuations. This ambiguity is due to the limited spatial resolution and detection efficiency. Cu and Zr were selected as model systems since the spatial distributions of the isotopes therein constitute ideal solid solutions, as the mixing enthalpy is, by definition, zero. Close agreement is observed between the simulated and measured spatial distributions of the isotopes. Having established the signature of a random distribution of atoms, the elemental distribution in amorphous Zr_59.3_Cu_28.8_Al_10.4_Nb_1.5_ samples fabricated by laser powder bed fusion is analyzed. By comparison with the length scales of spatial isotope distributions, the probed volume of the bulk metallic glass shows a random distribution of all constitutional elements, and no evidence for clustering is observed. However, heat-treated metallic glass samples clearly exhibit elemental segregation which increases in size with annealing time. Segregations in Zr_59.3_Cu_28.8_Al_10.4_Nb_1.5_ > 1 nm can be observed and separated from random fluctuations, while accurate determination of segregations < 1 nm in size are limited by spatial resolution and detection efficiency.

## 1. Introduction

Additive manufacturing (AM) enables fabrication of parts made from bulk metallic glass, circumventing limitations in the cooling rate of conventional techniques [1]. AM involves repeated heating and cooling, as a consequence of the layer-by-layer and laser-track-by-laser-track local periodic melting and solidification of the processed material [2]. For example, Zr-, Ti-, Fe- and Al-based bulk metallic glasses have been used for fabrication of components [3,4,5,6,7]. As the desired properties of metallic glasses stem from randomness in composition and structure [2], knowledge about the onset of clustering is essential from an application perspective. The here-studied alloy, Zr_59.3_Cu_28.8_Al_10.4_Nb_1.5_, is the composition of a widely used commercially available powder employed in laser powder bed fusion (LPBF) additive manufacturing. It is of particular interest as a model system as 35% of the published papers on processing Zr-based bulk metallic glasses by additive manufacturing techniques are focused on this composition [1]. The crystallization pathway of this alloy was studied by Pacheco et al. [8], where a cast reference was compared to a sample produced by LPBF. The authors concluded that the higher oxygen content of the LPBF sample resulted in a lower thermal stability, as they observed the Cu_2_Zr_4_O phase at 440 °C and a different initial phase formation. Metastable Cu_2_Zr_4_O nanocrystals were discovered already in the as-fabricated LPBF samples [8]. For the cast samples, the initial crystallization was observed at 480 °C with Al_2_Zr_3_ as an intermediate phase [8]. For both sample types, the crystallization resulted in CuZr_2_ and Al_3_Zr_4_ as the main phases present at 800 °C. The formation of both Cu_2_Zr_4_O and oxygen-rich α-Zr was observed by Marattukalam et al. in a study exploring the influence of different LPBF parameters on the crystallinity and mechanical properties for this composition [9]. α-Zr was observed by transmission electron microscopy (TEM) in an X-ray amorphous sample produced with a laser power of 65 W, while Cu_2_Zr_4_O was observed in a partly crystalline sample manufactured with 100 W [9]. Increased laser powers lead to larger melt pools as well as lower local cooling rates. The resulting temperature gradients in the melt pool and heat-affected zone cause a different degree of crystallinity and the presence of different crystalline phases [2]. Small-angle neutron scattering (SANS) was employed to study early stages of the crystallization process of LPBF samples in terms of population and cluster sizes, calculated using the difference in scattering length density of Al_3_Zr_4_ to the amorphous matrix, which is similar to that of the Cu_2_Zr_4_O phase [10]. Before heating, no evidence for the presence of clusters was obtained [10]. After isothermal annealing at 370 °C for 90 min, the clusters in the LPBF samples (75 W laser power) were calculated to have reached a mean radius of about 3 nm [10], which is 1–2 orders of magnitude smaller than the ones observed previously by TEM in the as-printed samples (65 and 100 W laser power) [9].

Phase separation has been studied for a wide range of compositions in binary Zr–Cu amorphous alloys [11] and Zr–Cu-based metallic glasses, for which both nanoscale decomposition in the amorphous state, e.g., [12,13,14] and nanocrystalline regions, e.g., [13,15], are reported. Nanoscale Cu clusters have been observed, for example, in a Zr–Cu–Al–Ni alloy after annealing below T_g_ [16]. While the size of these clusters challenges the resolution limits of most characterization techniques, atom probe tomography (APT) is suitable for spatially-resolved analysis of the elemental composition [17] and distribution [18] at the nm scale. This characterization technique is based on the combination of mass spectrometry and projection microscopy. Field evaporation [19] of atoms or molecules is assisted by application of a DC voltage and voltage or laser pulses to a needle-shaped specimen with a radius of tens of nanometers. Evaporated species are subsequently ionized up to charge states of +4 due to the surface electric field [20,21] and trajected towards a position-sensitive detector. The measurement of the time-of-flight allows for obtaining mass-to-charge state ratios of each detected ion or molecule. Spatial information is collected by the detector hit position of the ion, and projection algorithms are used to determine the original position of the atom or molecule within the specimen prior to field evaporation. The subsequent removal of species enables three-dimensional tomographic quantitative probing, and, in contrast to TEM, APT offers the advantage of a three-dimensional, spatially resolved nanoscale characterization. Eventually, clusters can be detected, which may or may not result in sufficient contrast using TEM [22]. The analysis of atomic scale clusters with sizes < 1 nm or below, albeit, remains challenging [23] as the aberrations during atom probe tomography imaging affect neighborhood relationships which are consequently not conserved after field evaporation and data reconstruction [23,24].

Various algorithms have been developed to identify clusters in APT data [25]. They are commonly based on comparing the measured data with a statistically random system or randomly swapped mass-to-charge-state ratios [23]. A typical assessment to find out whether a distribution is likely to be observed in a random arrangement of the ions can encompass the following steps [26]:

1. Visual inspection of the reconstruction and the use of isoconcentration surfaces.
2. Binomial analysis: Comparing the local composition in voxels of similar size with the same number of atoms to a binomial distribution and calculation of the Pearson correlation coefficient. For the Pearson correlation coefficient, the chi^2^ measure is normalized by sample size, and a value of 0 indicates a random distribution.
3. Nearest-neighbor analysis.
4. Cluster-finding algorithms.

However, all of these analysis protocols cannot give an unambiguous answer according to Cairney et al. [26]. The distinction of small clusters from random fluctuations is challenging in the context of APT due to the limited spatial resolution caused by trajectory aberrations [23], nonunity detection efficiency [18], and the reconstruction protocols [23]. The detection probability in APT does not relate to the Z number of the atom, but is a statistical process [27]. Detection is a limiting factor for studying small clusters, for example, Moody et al. [18] reported that the detection efficiency < 1 leads to an underestimation of the clustering. By considering a limited detection efficiency, it is evident that through random removal of atoms from segregations, the sample appears more random after data reconstruction than it is before field evaporation [18]. De Geuser and Gault [23] argue that the effective resolution limitation restricts analysis of small clusters < 1 nm and that many cases where detection has been discussed as a limiting factor are rather resolution-limited. APT resolution is well known to be anisotropic—due to trajectory aberrations, it is better in depth than laterally [27]—and is affected by a number of parameters, for example, experimental conditions such as temperature and electric field strength [27]. The depth resolution additionally strongly depends on the employed reconstruction protocol [23]. Trajectory overlaps lead to stochastic blurring, which in the case of small clusters result in atoms from the particles imaged within the matrix and vice versa [23]. This becomes increasingly critical the smaller the clusters are, and can affect the smallest apparent and, hence, resolvable size of objects [23]. These effects depend on the studied system, but as a general guideline, De Geuser and Gault established that clusters with a radius of <1 nm (∼250 atoms) cannot be accurately characterized, despite being detected [23].

In the present study, Zr_59.3_Cu_28.8_Al_10.4_Nb_1.5_ (herein referred to as Amloy) samples produced according to [9] are characterized by atom probe tomography to reveal potentially present nm-scale clusters in the as-processed samples. Heat-treated samples, comparable to the ones in which clustering was previously observed by Ericsson et al. [10], are measured as a reference. While the chemical homogeneity of Zr–Cu–Al metallic glasses has been predicted by molecular dynamics simulations [28], the experimental identification of nanoscale clusters is challenging. Therefore, we exploited the nanoscale chemical composition analysis by APT with a spatial resolution of ∼1 nm. To distinguish the onset of clustering from random fluctuations in the bulk metallic glass, we compare with a simulation which exhibits complete spatial randomness, and spatially resolve random distributions of naturally occurring isotopes in Cu and Zr measured by atom probe tomography.

## 2. Materials and Methods

### 2.1. Material Selection and Sample Preparation

Industrial-grade powder with the trade name AMLOY-ZR01 (Heraeus Holding GmbH, Hanau, Germany), referred to as Amloy, was processed by laser powder bed fusion (LPBF) with different laser powers in an EOS M100 (EOS GmbH, Munich, Germany), while keeping all other parameters constant (laser speed: 2000 mm/s, hatch spacing: 100 µm, powder layer thickness: 20 µm). The laser power for the different samples was set to 55, 75, and 85 W, respectively. Further process-related details can be found in [9]. The samples used for this study were cylinders of 8 mm diameter, which were sectioned at approximately 1 mm above the build plate. The atom probe tomography samples were prepared from polished surfaces of these cuts. Laser powers of up to 75 W resulted in X-ray amorphous samples [9]. To induce clustering comparable to [10], LPBF samples produced with 75 W were heat-treated. The samples were sealed in vacuum capillaries and introduced to a furnace preheated to 390 °C. To ensure cluster formation, a slightly higher temperature than the 370 °C in [10] was used, which is also closer to the glass transition temperature reported at 398 °C [29]. After annealing times of 1, 5, and 10 h, respectively, the samples were quenched in water and removed from the capillaries. Statistically random spatial distributions were obtained experimentally from the pure metals zirconium and copper (Thermo Fisher (Kandel) GmbH, Kandel, Germany) as constitutional elements of the Amloy composition: A Zr crystal bar piece (oxygen < 50 ppm, 99.5% (metals basis)) and a Cu slug piece (oxygen free, 99.995+%). The isotope ratios of Zr (^90^Zr, ^91^Zr, ^92^Zr, ^94^Zr, ^96^Zr) and Cu (^63^Cu, ^65^Cu) were used for assessing the randomness of these reference samples.

### 2.2. Atom Probe Tomography Measurements

Atom probe specimens were prepared from the above-specified samples by focused ion beam (FIB) techniques using a Helios Nanolab 660 (FEI, Hillsboro, OR, USA) dual-beam microscope. The preparation was carried out according to a standard protocol [30] and the final cleaning step was carried out with 5 kV and 40 pA. Laser-assisted APT measurements were conducted using a LEAP 4000X HR (Cameca, Madison, WI, USA). The field evaporation parameters for the different sample materials are summarized in Table 1. The IVAS 3.8.0 software (Cameca, Madison, WI, USA) was used for reconstruction, employing the shank angle reconstruction protocol. An overview of the individual measurements is provided in Table 2. Pearson correlation coefficients were calculated as a first measure of potential clustering with 50 ion bins.

### 2.3. Data Analysis and Simulation

Voxelization of the reconstructed APT data was performed using constant volumes, where the cube edge length was varied from 5 to 1 nm. Information about the elemental and ionic composition as well as the density were determined. In addition, simulations of Cu, Zr, and Amloy APT samples were performed, which allow for a comparison regarding the spatial ion distributions of the experimental data. Spatial positions of the ions were randomly sampled from a uniform distribution, mean density, and total ion count, and the composition of each simulation was chosen to match the respective experimental dataset. The isotopes constituting the elemental metals Zr and Cu were chosen to represent a random spatial distribution. Compositions for Cu and Zr were based on the experimental samples measured isotope composition, which was very close to the natural abundance. Therefore, the isotopes ^90^Zr, ^91^Zr, ^92^Zr, ^94^Zr, ^96^Zr and ^63^Cu, ^65^Cu were considered for the modeling and analysis in the same way that different elements are considered in the Amloy samples. For the analysis of the isotope distribution in Zr, only Zr^3+^ ions were considered due to the ambiguity related to ZrH complex ions for other charge states. No further modifications or assumptions were considered to ensure comparison with a known and truly random distribution of ions.

## 3. Results and Discussion

The uncertainty of the measured local isotope ratios or compositions depends on the measurement- and reconstruction-affected local density, i.e., the number of ions counted in each voxel. Consequently, the spatially resolved density is analyzed for the pure element samples and the metallic glass, which are presented in the Appendix A. Density variations exceeding the simulated local variations in the ion counts were observed for all samples, but more strongly for the crystalline single element samples. The isotope distributions of Zr (^90^Zr, ^91^Zr, ^92^Zr, ^94^Zr, ^96^Zr) and Cu (^63^Cu, ^65^Cu) were well distinguishable in the mass spectra obtained by atom probe tomography. Both isotope distributions constitute ideal solid solutions as the mixing enthalpy is zero.

**Figure 1:**
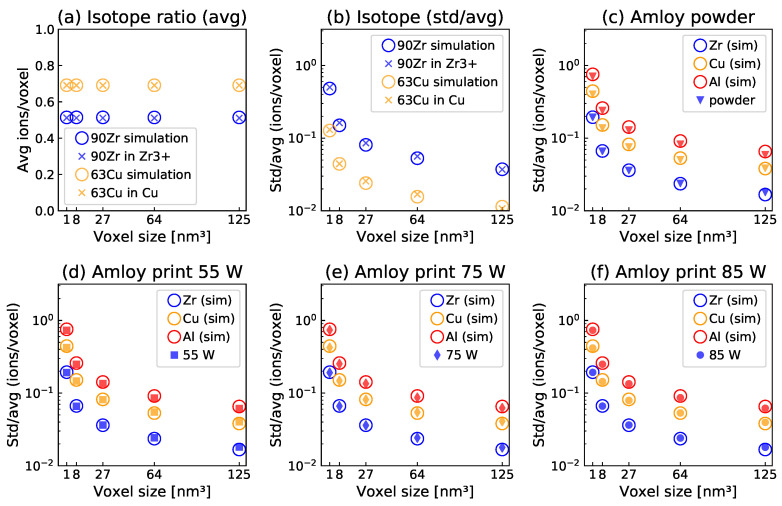
(**a**,**b**): Local ^90^Zr and ^63^Cu isotope distribution in pure zirconium and copper: mean (**a**) and std/avg (**b**) of the local isotope concentration versus voxel size in nm^3^. For Zr, only Zr^3+^ ions are considered due to ambiguity related to ZrH complex ions for other charge states. (**c**–**f**) APT measurements for Amloy powder (**c**) and printed parts (**d**–**f**). Std/avg for the local elemental concentrations of the main alloy components Zr, Cu, and Al versus voxel size in nm^3^.

To detect nanoscale clustering, the volume dependence of the respective isotope concentrations was sampled via fixed-size cubic subvolumes (voxels) ranging from 5 to 1 nm side length. Figure 1 shows the results of this analysis. The calculated mean abundances (isotope ratios, Figure 1a) from experimental data correspond well to the natural abundance of the respective isotopes and to the simulations, which are based on the reconstructed density and composition of the experimental data. The local isotope ratio variation is plotted as the standard deviation of the concentration of a selected isotope per voxel normalized by the mean concentration (std/avg) in Figure 1b. The very good agreement between measured and computed spatial isotope distributions underlines that both Cu and Zr indeed show ideal solid solution behavior and that a simulation exhibiting complete spatial randomness appears to adequately describe the measured data. These data serve as basis for comparison to the spatial elemental distributions measured for the metallic glass samples.

A similar analysis to that presented in Figure 1b is shown for the Amloy metallic glass samples produced with different LPBF parameters in Figure 1c–f. The mean composition for this alloy extracted from the APT measurements was Zr_63.4_Cu_25.0_Al_10.3_Nb_0.2_O_1.1_. The values of std/avg for the samples produced with laser powers from 55 to 85 W are in very close agreement with each other and with the simulations of random spatial distributions of this alloy. The comparison with the simulations, which are discussed in detail above, indicates a statistical, i.e., random distribution of all constitutional elements. The comparison between the samples in Figure 1 provides no evidence for clustering on the length scale probed with voxel sizes from 5^3^ to 1^3^ nm^3^. The drop in std/avg for increasing voxel sizes indicates the uncertainty associated with the number of sampled ions per voxel. This trend is also visible in the simulation of the alloy (shown as open circles in Figure 1). The close correspondence to the simulation illustrates that all samples appear random.

**Figure 2:**
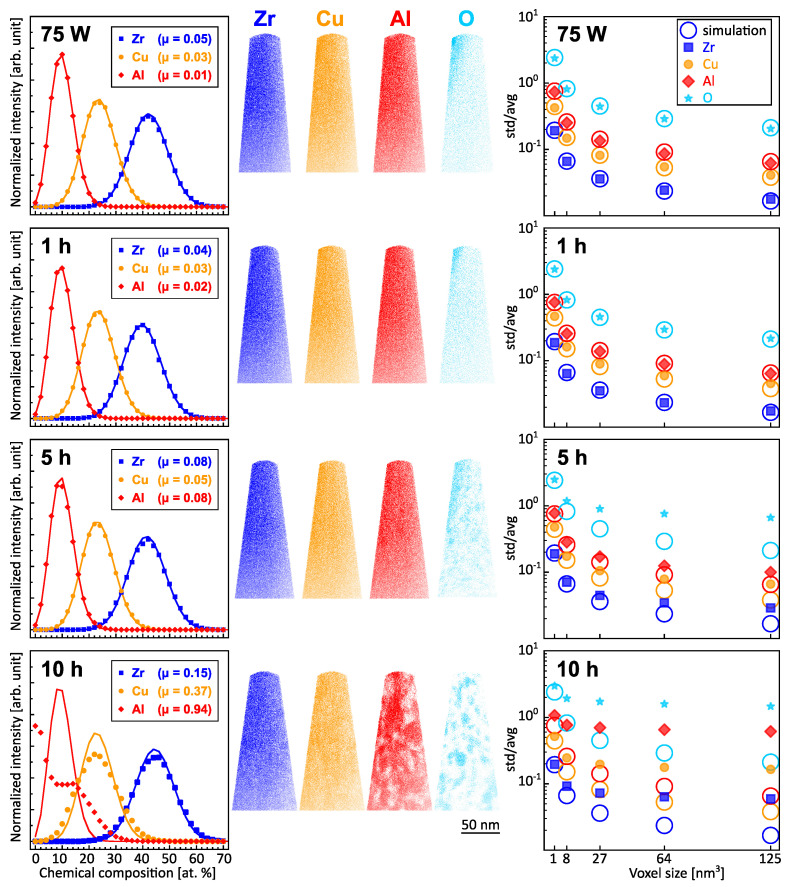
APT reconstructions of the samples processed with 75 W and annealed for 0, 1, 5, and 10 h at 390 °C. The frequency distribution analysis (calculated in IVAS 3.8.0 with bins of 50 ions) shows the concentration of the ions per voxel in comparison to a binomial distribution. Standard deviation (std) divided by average (avg) for the local elemental concentrations compared to the random simulation.

Previously, the effect of annealing on the thermal stability of LPBF samples produced with 75 W laser power was probed by SANS measurements, which indicated a fraction of clusters with around 2 nm radius present directly after a temperature of 370 °C [10]. After annealing at this temperature for 90 min, the clusters had grown to a mean radius size of around 3 nm [10]. Such features are, however, not visible in the LPBF samples presented here with a higher power parameter (85 W), which would correspond to a slower solidification than for lower powers due to larger melt pools. Hence, the LPBF processing exhibits a tolerance range for the metallic glass matrix, in which the increase in laser power does not result in the clustering observed in the previous annealing studies; instead, a homogeneous matrix is attained. To compare to such clustered samples, samples produced with 75 W were annealed under comparable conditions to those studied previously with SANS [10]. Visual inspection on the reconstructions shown in Figure 2 revealed clear clustering in the samples annealed for 5 and 10 h. The cluster sizes ranged around 2 nm in diameter for the 5 h sample and 5 nm for the 10 h sample. The formation of aluminum-rich clusters after annealing for 10 h is consistent with the diffusion of aluminum within a copper-rich matrix [31]. The as-printed and 1 h annealed samples exhibit an oxygen-rich region close to the surface of the tip, which corresponds to the surface of the sample. As these regions thus likely stem from surface oxidation, such regions are not included in the voxel analysis to determine clustering. The oxygen content of all atom probe specimens was 1.1 ± 0.1 at.%. Additional oxygen uptake was avoided due to vacuum capillary sealing of the samples before annealing, and the oxygen-rich regions, observed after 5 and 10 h of annealing, are therefore caused by redistribution of oxygen. With these samples, the voxel analysis presented above is tested in its capacity to detect these and possibly smaller clusters in the annealed samples.

The voxel analysis presented in Figure 2 confirms the visual inspection of the samples: For 5 and 10 h annealing time, a clear and increasing deviation from the simulation (marking a random distribution) is observed. For 1 h annealing, only a small shift for the standard deviations of Cu is observed, which is not clearly evident from the Pearson correlation coefficients µ. This confirms the growing clusters in the analyzed volume and strengthens the finding of a random distribution for the printed samples presented in Figure 1. Clusters on a length scale of few nm are thus detectable for the studied system. Furthermore, the voxel analysis (Figure 2) clearly reveals the clustering for the sample annealed for 5 h, while the Pearson correlation coefficients for this sample range only between 0.05 (Cu) and 0.08 (Zr, Al). The nanoscale clusters would thus not necessarily be distinguishable from the random sample (Pearson correlation coefficients of 0.01–0.05) if only this measure was analyzed. Clusters of >1 nm diameter were observed in the measurements and thus mark the minimum critical size under the employed measurement conditions. The heat treatments at 390 °C resulted in a clear trace of clustering from 5 h annealing time onwards and this was observed for Cu, Al, and O (Figure 2). However, nm-scale clusters were observed directly after the start of annealing in the SANS study [10], while even after one hour of annealing time at a slightly increased temperature (390 °C compared to 370 °C), no such clusters are observable here. SANS is thus more suited to detect the onset of clustering, while atom probe tomography can reveal the chemical nature of the clusters, which SANS cannot.

With the limited spatial resolution of APT, ordered materials may appear disordered in the reconstruction [32]. Depending on the material and analysis conditions, specific lattice planes may be visible, but in general, the atomic arrangement appears blurred due to resolution limitations, for example, the lattice positions of the pure metals analyzed above. For a random arrangement of different elements or isotopes, this resolution limitation has no further effect concerning the local concentrations, as shown in Figure 1. Small compositional features connected to the short- to medium-range order of the studied bulk metallic glass could, however, not be resolved in the reconstructions. The minimum cluster size observed ranged around 1–2 nm in diameter. As the resolution depends on many experimental factors, there is no general benchmark for which size such features can be excluded by their absence in the atom probe reconstruction. In the present study, the, by definition, random spatial distribution of isotopes in metals is used to compare between measured and simulated spatial randomness, on one hand, and to compare between measured random isotope fluctuations in pure Cu and pure Zr with the measured random or not random composition fluctuations in Amloy. A multi-element sample reconstructed as random does thus not necessarily imply a random distribution of the elements in the sample, but that such features were too small to be resolved. Consequently, additional techniques are necessary to complement the assessment of compositional fluctuations within small clusters by APT.

## 4. Conclusions

Nanoscale features can be chemically quantified by atom probe tomography. The challenge to resolve nm-scale compositional variations from random fluctuations arises from the limited spatial resolution, which is caused by trajectory aberrations, nonunity detection efficiency, and the employed reconstruction protocol. Trajectory aberrations are shown to cause density aberrations in the reconstructions of the tips. This effect is more pronounced for the crystalline elemental metals than for the amorphous alloy analyzed in this study. Elemental metals containing two or more isotopes constitute truly random spatial isotope distributions as their mixing enthalpies are zero. Therefore, we compare the magnitude of isotope fluctuations measured for ^63^Cu in Cu and ^90^Zr in Zr to the composition fluctuations in a Zr_59.3_Cu_28.8_Al_10.4_Nb_1.5_ sample processed by laser powder bed fusion. For the comparison to a respective random reference, simulations of all samples were performed, for which the atoms were distributed according to a random configuration. The close agreement between the experiments and the simulations strengthens the finding for the analyzed as-printed Zr_59.3_Cu_28.8_Al_10.4_Nb_1.5_ bulk metallic glass samples, in which no evidence for nanoscale clustering could be identified irrespective of the processing conditions. Thus, the fast quenching rates of laser powder bed fusion appear to suppress clustering, if compared to heat-treated samples where evidence for clustering is obtained. By comparison with the spatial isotope distributions of ideal solid solutions, cluster sizes of >1 nm in diameter were observed for annealed Zr_59.3_Cu_28.8_Al_10.4_Nb_1.5_ samples, marking the observable size range under the measurement conditions. Even though the presence of clusters < 1 nm in diameter can thus not be unambiguously excluded for the as-printed samples, clusters > 1 nm would have been reliably detected and can therefore be excluded. Further studies with complementary techniques, for example, transmission electron microscopy (TEM) and electron energy loss spectroscopy (EELS), can be used to provide correlation between crystalline phases and the local atomic composition.

## Figures and Tables

**Table 1:** Atom probe tomography measurement parameters.

Material	Laser Pulse Energy	Laser Pulse Frequency	Base Temperature	Detection Rate
	[pJ]	[kHz]	[K]	[%]
Zr	10	125	30	0.5
Cu	10, 100	125	30	0.5
Amloy	60	200	60	0.5

**Table 2:** Atom probe tomography measurements overview. Bulk Amloy samples were produced by LPBF with different laser power settings. An annealing temperature of 390 °C was used for the heat treatments.

Material	Processing	Sample Population [×10^6^ ions]
Zr	as-received	1.6
Cu	as-received	1.6
Amloy	as-received powder	11.0
Amloy	LPBF—55 W	10.9
Amloy	LPBF—75 W	7.9
Amloy	LPBF—85 W	12.0
Amloy	LPBF—75 W + annealed 1 h	7.7
Amloy	LPBF—75 W + annealed 5 h	8.1
Amloy	LPBF—75 W + annealed 10 h	9.7

**Figure A1:**
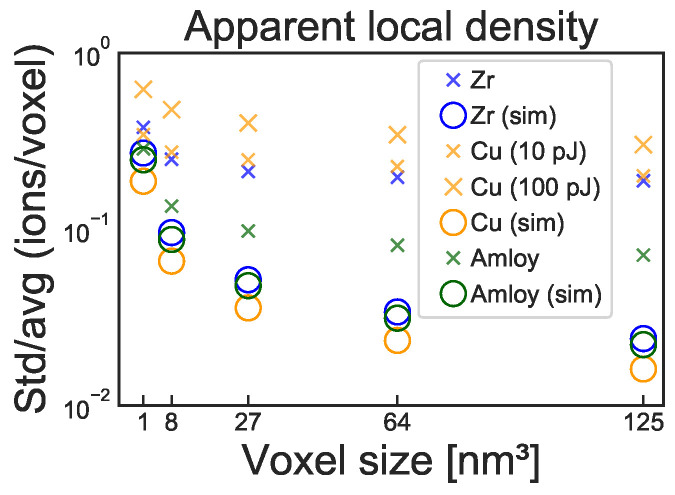
Reconstructed apparent local density variations (expressed as standard deviation divided by mean) for the different sample types and voxel sizes.

## Data Availability

The data supporting the findings of this study are available from the corresponding author upon reasonable request.

## Appendix A. Local Reconstructed Density

The local reconstructed density is expected to fluctuate due to the random detection. For comparison to the measured data, a random density distribution is calculated (see Section 2). A broadening of the experimentally obtained respective density distribution beyond the simulated, expected contribution can be attributed to trajectory aberrations or the reconstruction protocol. Trajectory aberrations occur due to the complex evolution of the field evaporating surface, which leads to a varying specimen shape in the course of the measurement and thus a varying magnification [33]. For a homogeneous material, a steady-state shape is reached during the measurement; however, most APT studies focus on heterogeneous materials, which induce a variation of the reconstructed density [33]. These effects are well studied for cases of multiphase materials and crystallographic features, leading to locally different magnifications and thus reconstructed density fluctuations [34].

For the present samples, a distinctive difference is observed in the reconstructed local densities. They vary for all samples more strongly (indicated by a higher standard deviation) than expected from the simulation, as shown in Figure A1. For the metals Zr and Cu, these variations on a length scale of a few nm are very unlikely to have any physical origin. The recorded difference can thus be attributed to the measurement conditions. The simulation is based on a statistically random removal of atoms due to the detection efficiency. The difference in the simulations shown in Figure A1 is caused by the different mean densities of the APT reconstructions, which vary between 13.7 ions/nm^3^ for Zr and 15.8 ions/nm^3^ for the Amloy composition to 28.5 ions/nm^3^ for Cu. The offset observed between the APT measurement and this simulation therefore corresponds to the order of magnitude of trajectory aberrations in the projection of the tip. For Cu, different laser pulse energies were analyzed and have a clear influence: with higher thermal energy input (100 pJ compared to 10 pJ) and, thus, increased temperatures, the magnitude of the reconstructed density variations increases, probably due to increased surface mobility of the ions prior to departure. Gault et al. [27] found that increasing the temperature does not necessarily have a strong effect on the resolution below a certain threshold, above which the effect then becomes pronounced. The present result indicates that the 100 pJ measurement exceeded this threshold. The offset is distinctly different for the studied systems: for the pure metals analyzed with 10 pJ laser energy the effect is comparable, while it is much less pronounced for the Amloy samples, most likely due to the amorphous nature of these samples, and may consequently be related to the absent effect of the formation of crystalline terraces, where the atoms at the side are evaporated preferentially [27].
